# Label-Free Monitoring of Diffusion in Microfluidics

**DOI:** 10.3390/mi8110329

**Published:** 2017-11-09

**Authors:** Kristian Tølbøl Sørensen, Anders Kristensen

**Affiliations:** Department of Micro- and Nanotechnology, Technical University of Denmark, Kongens Lyngby 2800, Denmark; kriss@nanotech.dtu.dk

**Keywords:** optofluidics, microfluidics, diffusion, convection, mixing, refractive index, label-free

## Abstract

Label-free, real-time detection of concentration gradients is demonstrated in a microfluidic H-filter, using an integrated photonic crystal slab sensor to monitor sample refractive index with spatial resolution. The recorded diffusion profiles reveal root-mean-square diffusion lengths for non-fluorescing and non-absorbing molecules, both small (glucose, 180 Da) and large (bovine serum albumin, 67 kDa).

## 1. Introduction

Microfluidics have become a standard solution to challenges involving accurate fluid control and small sample volume [1], as the laminar flow conditions often employed in microfluidics ensure mixing of compounds only by molecular diffusion. Such processes can be monitored easily when liquids are visible, but may leave the experimenter blind when using non-absorbing and non-fluorescing compounds. In these cases, the effects of flow adjustments may perhaps only be recognized after off-line analysis.

Depending on its application, the microfluidic structure employed in this paper is referred to as an H-filter [2,3], typically when its purpose is to filter smaller molecules from larger particles, or a T-sensor, when its purpose is to monitor the diffusion and interaction of analytes [1,4]. Such devices have been used to investigate, e.g., protein binding [5], chemical extraction [6,7], blood dialysis [8], and for membrane-less fuel cells [9]. Devices for flow injection analysis [10], general flow focusing [11], and spiral microfluidics [12] are also closely related, and the ability to monitor the extent of diffusion is also highly relevant to those applications. In general, molecular diffusivity is a central physical property, governing chemical reaction kinetics and mass transfer phenomena, which are often at the core of microfluidic architectures.

A photonic crystal slab (PCS) sensor [13], also referred to as a guided mode resonance filter [14], leaky-mode resonant reflector [15], or resonant waveguide grating [16] is an optical sensor, which responds to changes in refractive index (RI) within the first few hundred nanometers from its surface. Such RI sensors are universal, in the sense that they neither require compounds to be conducting, absorbing nor fluorescing. The fact that this class of sensors operate label-free is of convenience in many applications, as the process of labelling may not always be trivial, and for diffusion monitoring this is of critical importance, as the label itself would affect the very property under investigation. PCS sensors have been demonstrated for, e.g., protein binding affinity [17] and kinetics [18], and cell culture monitoring [19]. They are generally realized by deposition of a high-RI dielectric material such as titanium dioxide [20] or a suitable polymer [13] onto a sub-wavelength grating, which may be produced by nanoimprint-lithography into a polymer [21].

**Figure 1:**
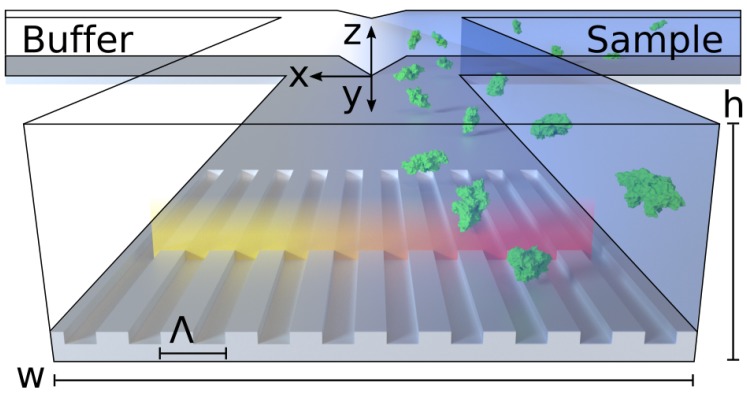
Schematic illustration of the sensor-embedded microfluidic H-filter operating principle. Two liquid streams meet and co-flow laminarly in a channel of height *h* and width *w*. The embedded sensor has a grating period of Λ and measures along a line. The color of light reflected by the sensor redshifts as molecules diffuse along *x*. Small molecules, shown in blue, will diffuse further and thus have a softer transition profile than larger molecules, exemplified by bovine serum albumin (BSA) in green (crystal structure 4F5S) [22]. The embedded label-free sensor monitors the extent of diffusive mixing in real-time.

Here, we report an optofluidics system for universal detection of concentration gradients and monitoring of diffusion. As a proof-of-concept, we demonstrate a PCS sensor-embedded microfluidic H-filter, comprised entirely of polymers, illustrated conceptually in Figure 1. The H-filter is a common and versatile microfluidic component which separates compounds by size. We show that this optofluidic device allows label-free, real-time resolution of concentration gradients, both for small molecules and large proteins. To our knowledge, this is the first demonstration of real-time diffusion monitoring of non-absorbing, non-fluorescing species flowing in a microfluidic H-filter.

## 2. Theory

To avoid convective mixing through turbulence, it is a critical requirement that the H-filter be operated under laminar flow conditions, e.g., at a low Reynolds-number, $Re=\rho \bar{v}h/\eta$, where ρ is the fluid density, $\bar{v}$ is the average flow velocity and η is viscosity. For the channel dimensions and flow rates employed in this paper, the channel height *h* is the characteristic length, and the Reynold’s number in water becomes Re<0.1, ensuring strictly laminar flow. Under these conditions, the only way for a molecule to move across the channel is by diffusion.

Einstein showed that the root-mean-square displacement of a molecule undergoing Brownian motion, in the following referred to as diffusion length $\bar{x}$ (see Figure 1 for coordinate system), is given by $\bar{x}=\sqrt{2Dt}$, where *D* is the mass diffusivity and *t* is the diffusion timescale.

Fick’s second law relates the change in concentration (ϕ) over time to change in concentration over space (*x*) for a diffusing analyte: $\delta \phi /\delta t=D\delta^{2}\phi /\delta x^{2}$, which in one dimension has the solution:

(1) $$\phi \left(x\right)=\phi_{0}\mathrm{erfc}\left(\frac{x}{2\sqrt{Dt}}\right)=\phi_{0}\mathrm{erfc}\left(\frac{x}{\sqrt{2}\bar{x}}\right)$$

where $\phi_{0}$ is the concentration originally in the sample stream. Fitting this equation to an observed diffusion profile directly reveals the diffusion length $\bar{x}$.

Due to the parabolic flow profile causing molecules near the walls to move at a lower velocity, molecules near the walls are able to diffuse further across the channel than molecules found closer to the center of the channel, where the flow velocity is highest. This effect was named the butterfly effect by Kamholz et al. [5] due to its shape, and it allows an increased diffusion length for molecules advecting near a wall. However, this gradient evens out when the advective transport rate is low compared to the diffusive transport rate, a relationship described by the Péclet-number $Pe=\bar{v}h/D=Q/\left(wD\right)$, where *Q* is the volumetric flow rate and *w* is the channel width. Diffusion length $\bar{x}$ is independent of *z* when y/h≫Pe≫1 [23]. This is important for the measured diffusion length to be representative for the whole channel, as the PCS sensor measures the concentration gradient within the first few hundred nanometers from the sensor wall.

For pressure-driven flow, the relationship between pressure gradient Δp, flow rate *Q* and hydraulic resistance $R_{\mathrm{hyd}}$ is given by the Hagen-Poiseuille law [24] $\Delta p=QR_{\mathrm{hyd}}=12\eta LQ/\left(h^{3}w\right)$, where *L* is the length of the wide and shallow channel (h≪w).

## 3. Method

**Figure 2:**
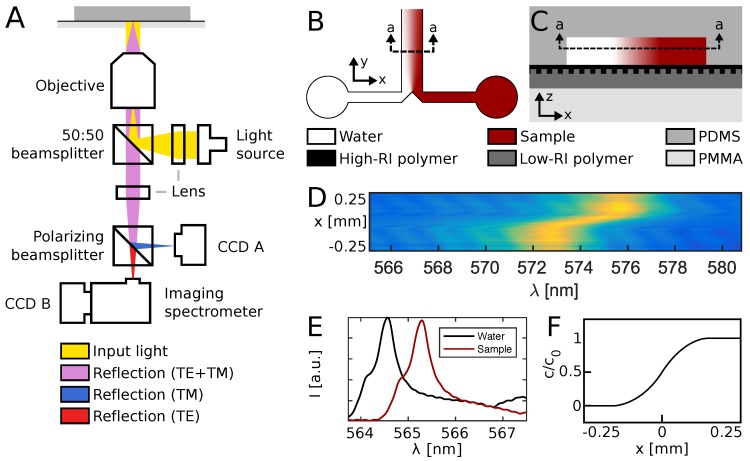
Setup and device operation. (**A**) Schematic overview of optical components. Light reflected by the sensor is received by an imaging spectrometer and used to monitor refractive index changes. (**B**) Top view of the microfluidic system. Two liquid streams co-flow and mix only by diffusion. (**C**) Side view of the sensor-embedded microfluidic H-filter, comprised by four different polymers. (**D**) Sample spectral image showing the concentration gradient. (**E**) Resonance peaks at two positions. The presence of analyte red-shifts the resonance wavelength. (**F**) Diffusion profile as revealed by the embedded sensor.

Figure 2A shows the optical system used to read out the optofluidic device, as previously described [13]. Briefly, the setup features a bright and spectrally flat, white light source (EQ-99X LDLS, Energetiq Technology, Inc., Woburn, MA, USA), directed through a lens, beamsplitter and microscope objective to ensure illumination of the sensor wafer at normal incidence. Reflected light was guided back through the microscope objective and beamsplitter, and directed into an imaging spectrometer (Acton SP2750, Princeton Instruments, Trenton, NJ, USA) by a mirror, a tube lens and a polarizing beamsplitter. This setup ensured that only resonantly reflected light of transverse electric (TE) polarization reached the spectrometer, while the transverse magnetic (TM) light component was received by a camera CCD (Thorlabs, Newton, NJ, USA) and used for navigation. The resonance wavelength relates to the fluid refractive index [20], which is proportional to concentration (in *w*/*v* % [25]).

Microfluidic devices, illustrated in Figure 2B, were produced by casting of polydimethylsiloxane (PDMS, Sylgard 184, Dow Corning, Midland, MI, USA) on a silicon stamp to a thickness of approx. 10 mm, followed by a 2-h curing at 80 °C. The stamp was produced by standard UV photolithography into a 9 μm thick AZ-4562 resist film, using a custom photomask (Delta Mask, Enschede, The Netherlands). The mask defined channels of w=1 mm width and L=20 mm length, with a channel height h=9 μm as defined by the resist thickness. This height was verified by profilometry and Fabry-Pérot interference analysis. Before using the silicon stamp for PDMS casting, a perfluorodecyltrichlorosilane coating was applied by molecular vapor deposition. Inlet holes were punched using a 0.5 mm diameter biopsy punch (World Precision Instruments Ltd., Sarasota, FL, USA), and the microfluidic device formed a strong and irreversible bond upon contact with the sensor wafer, after the two parts had been exposed to 60 s of oxygen plasma at 150 W.

The all-polymer sensor wafer was fabricated as described by Hermannsson et al. [13]. Briefly, a low-RI polymer, Efiron PC-409AP (Luvantix ADM, Daejeon, Korea), was diluted to 85% in 2-propanone (Sigma Aldrich, St. Louis, MI, USA), and UV nanoimprinted on a 2 mm thick poly(methyl methacrylate) (PMMA) substrate, using a silicon stamp defined by electron beam lithography. The stamp featured 4 × 4 fields, each of 2 mm × 2 mm, spaced 9 mm apart, with a grating period of Λ=368 nm and a grating depth of d=100 nm. A high-RI polymer, HI01XP (Micro Resist Technology GmbH, Berlin, Germany), was diluted to 25% in ma-T 1050 (Micro Resist Technology GmbH) and spincoated at 3000 rpm for 60 s to form a homogenous, conformal layer. After at 3-min bakeout at 90 °C, the wafer was UV flood exposed in a nitrogen atmosphere for 5 min. Figure 2C illustrates the polymer stack comprising the optofluidic system after bonding.

Liquids were driven using a 4-channel digital pressure controller (MFCS Flex, Fluigent, Inc., Villejuif, France). Channels were first wetted using buffer, which for the case of glucose was merely MilliQ water, or phosphate buffered saline (PBS, Sigma Aldrich) for the case of bovine serum albumin (BSA, Sigma Aldrich). In the case of BSA, a full surface passivation was first performed, by leaving a 30 mg/mL solution in the channels for 30 min before starting the experiment.

## 4. Results and Discussion

A spectral image, such as the one illustrated in Figure 2D, was acquired every second, with a spectral resolution of 12 pm and a spatial resolution of 5 μm. The resonance wavelength $\lambda_{r}$ appears as a bright peak at a given *x*-position, and was calculated as the weighted average $\lambda_{r}\left(x\right)=\sum \lambda_{i}I_{i}/\sum I_{i}$, after values of $I<0.5(I_{\max}+I_{\min})$ had been set to zero. Resonance peaks at two representative positions are shown in Figure 2E. By probing the resonance peak at all 100 positions monitored simultaneously by the imaging spectrometer, a diffusion profile of the shape illustrated in Figure 2F was obtained every second.

Due to the no-slip boundary condition, at the stagnation point y=0 where the two streams meet, the flow velocity $v_{y}$ will be zero. At a certain distance $y_{L}$ known as the entrance length, the normal parabolic flow profile will have developed to 99%. It is thus important that measurements be made well downstream from $y_{L}$, but as it is typically on the order of 100 μm [5], this is rarely a problem in practice. All measurements were performed at $y_{\mathrm{glucose}}=19.1$ mm and $y_{\mathrm{BSA}}=18.1$ mm for the two sample liquids, thus avoiding entrance effects.

**Figure 3:**
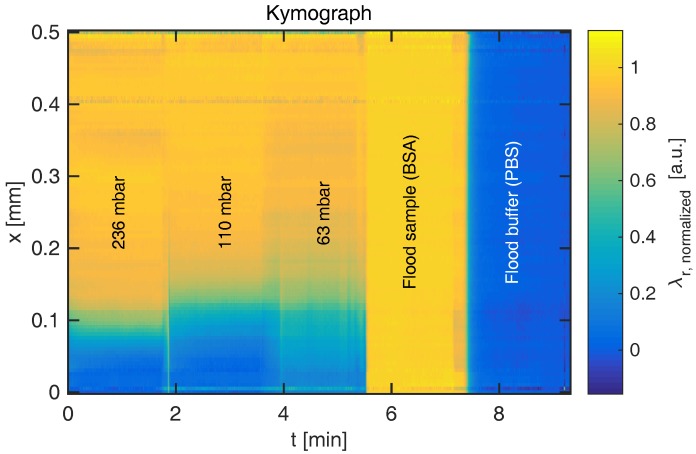
Kymograph of BSA diffusion experiment, obtained by end-to-end fusing the resonance wavelength distribution for each frame. The color corresponds to resonance wavelength shift normalized to the reference values in pure PBS buffer ($\lambda_{r}=0$) and pure BSA ($\lambda_{r}=1$). The sharpness of the transition increases with measured pressure.

**Figure 4:**
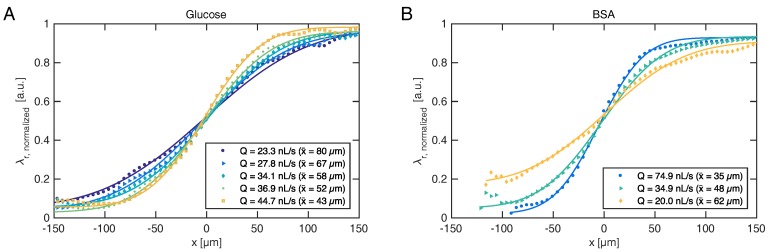
Experimental diffusion profiles for (**A**) glucose and (**B**) BSA. Higher flow rates (*Q* in the legends) lead to sharper transition (smaller $\bar{x}$) at a given downstream position. Solid lines indicate best fits to Equation (1).

The resonance wavelength distribution $\lambda_{r}\left(x\right)$ of each frame can be combined to form a kymograph displaying time on the first axis, as shown in Figure 3. It is evident that the RI boundary becomes sharper when the pressure difference increases, which decreases the time for diffusion and thus diffusion length. The figure also indicates the reference signals when the channel is entirely filled with sample or buffer, respectively. As the sharp boundaries on the temporal axis indicate, steady state is rapidly achieved. At the lowest flow rate shown here (Q=19.1 nL/s), the convection time is $t_{\mathrm{conv}}=ywh/Q=9.15$ s. Following a change in flow rate, a delay of $t>t_{\mathrm{conv}}$ should thus be permitted to ensure steady state. By temporal averaging of the resonance wavelengths within each section, steady-state diffusion profiles are obtained for various flow rates, as shown for glucose and BSA in Figure 4.

Fitting Equation (1) to the experimentally obtained diffusion profiles directly reveals diffusion length $\bar{x}$, which governs the separation efficiency of the H-filter. The ability to monitor the extent of diffusion in real-time allows the operator to adjust flow conditions during an experiment, rather than waiting for results of a subsequent off-line analysis to determine whether the conditions were in fact suitable. Often such adjustments must otherwise be made “in the dark” for real samples, which may neither be absorbing nor fluorescing, as usually the diffusion boundary cannot be visualized.

For complex samples, such as mixtures of protein fragments, each different component would exhibit a size-dependent diffusion, and the measured refractive index profile would comprise a superposition of multiple error functions (Equation (1)). An analysis of the concentration gradient would reveal an average diffusion length, which may be sufficient for certain applications.

To summarize, a PCS sensor-embedded microfluidic H-filter was presented, which enables label-free, real-time monitoring of concentration gradients. As a proof of concept, the device was used to monitor diffusion of glucose and BSA, and the diffusion length in response to various flow rates was determined. This upgrade to a classic microfluidic component could enable many new applications previously precluded by the need for samples to be fluorescing or absorbing. The demonstrated sensor-integrated optofluidic device enables informed decision-making on the basis of diffusion-length, which could further act as input for an automated feedback loop. In this work we focus on measuring diffusion length. This could be extended to determining diffusion constants. However, for high-viscosity samples, the mutual diffusion coefficient would obtain a spatial dependence, which could be the topic of future investigations.
